# The Flp type IV pilus operon of *Mycobacterium tuberculosis* is expressed upon interaction with macrophages and alveolar epithelial cells

**DOI:** 10.3389/fcimb.2022.916247

**Published:** 2022-09-20

**Authors:** Christopher J. Alteri, Nora Rios-Sarabia, Miguel A. De la Cruz, Jorge A. González-y-Merchand, Jorge Soria-Bustos, Carmen Maldonado-Bernal, María L. Cedillo, Jorge A. Yáñez-Santos, Ygnacio Martínez-Laguna, Javier Torres, Richard L. Friedman, Jorge A. Girón, Miguel A. Ares

**Affiliations:** ^1^ Department of Natural Sciences, University of Michigan-Dearborn, Dearborn, MI, United States; ^2^ Unidad de Investigación Médica en Enfermedades Infecciosas y Parasitarias, Hospital de Pediatría, Centro Médico Nacional Siglo XXI, Instituto Mexicano del Seguro Social, Mexico City, Mexico; ^3^ Departamento de Microbiología, Escuela Nacional de Ciencias Biológicas, Instituto Politécnico Nacional, Mexico City, Mexico; ^4^ Instituto de Ciencias de la Salud, Universidad Autónoma del Estado de Hidalgo, Hidalgo, Mexico; ^5^ Laboratorio de Investigación en Inmunología y Proteómica, Hospital Infantil de México Federico Gómez, Mexico City, Mexico; ^6^ Centro de Detección Biomolecular, Benemérita Universidad Autónoma de Puebla, Puebla, Mexico; ^7^ Centro de Investigación en Ciencias Microbiológicas, Benemérita Universidad Autónoma de Puebla, Puebla, Mexico; ^8^ Department of Immunobiology, University of Arizona, Tucson, AZ, United States

**Keywords:** tuberculosis, mycobacteria, pili, adherence, virulence, biofilms

## Abstract

The genome of *Mycobacterium tuberculosis* (*Mtb*) harbors the genetic machinery for assembly of the Fimbrial low-molecular-weight protein (Flp) type IV pilus. Presumably, the Flp pilus is essential for pathogenesis. However, it remains unclear whether the pili genes are transcribed in culture or during infection of host cells. This study aimed to shed light on the expression of the Flp pili-assembly genes (*tadZ, tadA, tadB, tadC, flp, tadE*, and *tadF*) in *Mtb* growing under different growth conditions (exponential phase, stationary phase, and dormancy NRP1 and NRP2 phases induced by hypoxia), during biofilm formation, and in contact with macrophages and alveolar epithelial cells. We found that expression of *tad/flp* genes was significantly higher in the stationary phase than in exponential or NRP1 or NRP2 phases suggesting that the bacteria do not require type IV pili during dormancy. Elevated gene expression levels were recorded when the bacilli were in contact for 4 h with macrophages or epithelial cells, compared to mycobacteria propagated alone in the cultured medium. An antibody raised against a 12-mer peptide derived from the Flp pilin subunit detected the presence of Flp pili on intra- and extracellular bacteria infecting eukaryotic cells. Altogether, these are compelling data showing that the Flp pili genes are expressed during the interaction of *Mtb* with host cells and highlight a role for Flp pili in colonization and invasion of the host, subsequently promoting bacterial survival during dormancy.

## Introduction

*Mycobacterium tuberculosis* (*Mtb*) is the bacteria responsible for causing tuberculosis, a highly prevalent infectious and devastating disease worldwide. It is estimated that 10.0 million new cases and 1.5 million deaths due to tuberculosis occurred in 2020 (World Health Organization, 2021). *Mtb* is an intracellular organism that interacts with alveolar macrophages in the niche of the lower respiratory tract, where it can reside for a long time in a susceptible host. Despite many years of research and elucidating the genome sequence of *Mtb* strains, we still do not know much about its pathogenesis. Understanding what bacterial and host factors are essential for pathogenesis is critical to unraveling novel therapeutic approaches to prevent or treat tuberculosis.

The pathogenicity of intracellular microorganisms is a complex multifactorial process that largely depends on their ability to colonize mucosal surfaces, invade, multiply, and damage eukaryotic cells and tissues, and interfere with the host’s defense mechanisms (Kline et al., 2009). Bacteria utilize fimbrial and non-fimbrial adhesins to interact with each other during biofilm formation and in the colonization of host tissues. For many years, *Mtb* was regarded as a non-piliated organism until Alteri et al., identified for the first time in *Mtb* strain H37Rv a pilus structure called *Mtb* pilus (MTP), whose expression was influenced by nutritional conditions and environmental signals (Alteri et al., 2007). MTP are flexible, thin (2–3 nm in diameter), and highly aggregative fibers composed of major pilin subunits encoded by the *mtp* gene (Rv3312A). MTP shared some of the biochemical properties of amyloid fibers, which have been found to be associated with other human diseases. IgG antibodies reactive with MTP were detected in sera obtained from patients undergoing active tuberculosis, indicating that the pili are produced *in vivo* during host infection (Alteri et al., 2007). No anti-MTP antibodies were found in non-tuberculosis patients. MTP bind the host extracellular matrix protein laminin. Antibodies against MTP detected the fibers in *Mtb*’s bacterial aggregates, suggesting that the pili promote bacterial-bacterial interactions and biofilm formation. These compelling data support a crucial role for MTP in the biology of the bacteria within the host. Furthermore, deletion of Rv3312A in strain V9124 yields an MTP-mutant that was defective in biofilm formation, adherence, and invasion of A549 alveolar epithelial cells (Ramsugit et al., 2013; Ramsugit et al., 2016). However, the mutant could still adhere and invade THP-1 macrophages (Ramsugit and Pillay, 2014).

Type IV pili are proteinaceous surface-exposed polymeric appendages produced by a large number of bacterial pathogens for humans, plants, and animals. Type IV pili perform a variety of functions associated with pathogenicity, such as promoting bacteria-bacteria interactions, biofilm formation, twitching motility, binding of bacteriophages, DNA uptake, and cell adherence favoring interactions of pathogens with their corresponding epithelial and phagocytic cells (Craig et al., 2004). Type IV pilins share several biochemical features; generally, they are highly hydrophobic and conserved. There is an invariable glutamate residue in the fifth position of the mature pilin amino terminus and a conserved glycine residue preceding the cleavage site by the prepilin peptidase (Craig et al., 2004). Based on the biochemical properties of the pilins, the type IV pili family of proteins has been classified into Class A and B (Craig et al., 2004). The Tight Adherence (Tad) or Fimbrial Low-molecular-weight *P*rotein (Flp) type IVB pili were first described in *Actinobacillus actinomycetemcomitans*. They were associated with rough colony appearance and their ability to autoaggregation and form biofilms (Kachlany et al., 2000). The Flp pili are widely distributed across genera and species, including *Mycobacteria* (Mann et al., 2016). Bacterial pathogens such as *Bordetella pertussis* harbor two distinct *tad* loci, whereas others, including *Mesorhizobium loti*, *Burkholderia pseudomallei*, and *Vibrio vulnificus*, contain three copies. The *flp* gene encodes the Flp major type IV pilin subunit (Tomich et al., 2007).

Analysis and bioinformatic inquiry of the genome sequence of *Mtb* H37Rv seeking potential pilin homologs led to the discovery of a seven-gene cluster (*tadZ, tadA, tadB, tadC, flp, tadE*, and *tadF*), which has been thought to participate in biosynthesis and assembly of Flp. However, no information on gene expression or production of type IV pili in *Mtb* is available. The *tadZ* gene encodes a protein that directs the cell location of the pilus. The *tadA* gene codes for an ATPase, while the *tadB* and *tadC* genes code for two membrane proteins that assemble the pilus. *tadE* and *tadF* code for minor pilins. Downstream from the pilus operon lies the *pilD* gene, which encodes a pre-pilin peptidase responsible for processing the prepilins into mature pilins (Kachlany et al., 2001a; Schreiner et al., 2003; Danelishvili et al., 2010). The contribution of the Flp to the virulence-associated properties of *Mtb* is mainly unknown. In the present study, we sought to evaluate the expression of Flp type IV pili genes under different growth conditions and during the interaction of the mycobacteria with macrophages and alveolar epithelial cells adherence. This study advances our knowledge of the *Mtb* mechanisms of bacteria-bacteria and bacteria-host cell interactions and identifies a new target for vaccine development against tuberculosis.

## Materials and methods

### DNA and protein sequences analysis

The National Center for Biotechnology Information BLAST server program was used to search for DNA sequences encoding type IV pili and determine protein homology. Clustal W was employed to align predicted protein sequences. DNA sequences’ overall and G+C % content were analyzed by scanning regions of *Mtb* H37Rv genome sequence with a 40-base sliding window using Vector NTI software Bio-Plot functions. Additional G+C determinations were done using the Z curve database applications at (http://tubic.tju.edu.cn/zcurve/) (Zhang et al., 2001). REPFIND (http://zlab.bu.edu/repfind/) is a bioinformatic tool to detect clustered and exact repeats in nucleotide sequences. To remove repeat sequences occurring by chance, REPFIND was used with a P-value cut-off of 0.0001, and the query sequence was employed to determine the statistical background.

### Bacterial strains and culture conditions

*Mtb* H37Rv was grown in Dubos (Difco) culture medium complemented with 10% of the mixture albumin, dextrose, and catalase (ADC; BBL) and incubated at 37°C. Bacteria were harvested at exponential and stationary phases when they reached an OD_600_ of 0.4 and 1.2, respectively. For cell infections, the bacterial inoculum was prepared as follows: *Mtb* was grown in Middlebrook 7H9 (Difco) supplemented with 10% ADC (BBL) and 0.05% Tween-80 (Sigma) and incubated at 37°C until an OD_600_ of 1.0 was reached.

### Hypoxia conditions

Aerobic culture of *Mtb* H37Rv growing at the exponential phase in a sealed roller flask with a headspace ratio of 0.5 was subjected to hypoxia as described previously (Wayne and Hayes, 1996); Hypoxia was monitored in a parallel culture by discoloration of the redox indicator methylene blue (1.5 µg/mL). Non-replicative persistence 1 (NRP1) was attained at five days of exposure to hypoxia (partial discoloration of the dye) and non-replicative persistence 2 (NRP2) at 15 days (complete discoloration of the dye).

### Biofilm formation

Biofilm assays were performed using a modification of Ojha et al. (2008). Mycobacterial cells from the stationary phase (OD_600 =_ 1.2) were diluted at 1:100 in a new Dubos medium with 10% ADC, and 2 mL were added to each well in a six-well flat-bottomed polystyrene plate (Costar). The plate was covered and wrapped with parafilm and incubated at 37°C in 5% CO_2_ for five weeks (Ojha et al., 2008). The planktonic cells’ supernatants were transferred to a new tube for posterior centrifugation and RNA extraction. After the biofilms were washed thrice with sterile PBS and dried in a biosafety cabinet, the biofilm cells were suspended in DEPC water and scraped with a pipette. The cell suspension was centrifuged at 8,000 x *g* for 5 min. RNA was extracted from the cell pellets for gene expression experiments as described below.

### Cell lines and culture conditions

The human monocytic U-937 (ATCC CRL-1593.2) and human alveolar epithelial basal A549 (ATCC CCL-185) cell lines were used in this study. The cells were grown in RPMI-1640 (Gibco) supplemented with heat-inactivated 10% fetal bovine serum (FBS, Gibco) in 75 cm^2^ tissue culture flasks at 37°C under an atmosphere of 5% CO_2_ (Wei et al., 2000). Cell monolayers at 80% confluence were seeded into 24-well culture plates as needed in subsequent experiments. U-937 monocytes were treated with 200 nM of phorbol 12-myristate 13-acetate (PMA, Sigma) for 24 h to promote differentiation into macrophages; then, monolayers were replenished with a new medium.

### Gene expression upon infection of cultured phagocytic and epithelial cells

Semi-confluent (80%) monolayers of U-937 and A549 cell lines (1 x 10^4^ cells/well) were incubated with 10 μL of *Mtb* H37Rv (10^8^ bacteria/mL) at 37°C under 5% CO_2_ atmosphere. Bacteria exposed to RPMI-1640 medium for 4 h were used as control. After infection, the mycobacteria in the supernatant were collected to determine whether the microenvironment generated by the interaction with the cells influenced the expression of type IV pili genes. Bacterial RNA from control and experimental samples were then extracted as described below.

### RNA isolation and preparation of cDNA

RNA isolation was carried out as described before (Rustad et al., 2009). Briefly, mycobacteria cultures obtained at different growth phases (exponential, stationary, NRP1, and NRP2 stages of hypoxia), planktonic, biofilm bacteria, and bacteria from cell infections were resuspended in 1 mL of Trizol (Sigma) and transferred to screw-cap tubes containing ~0.5 mL of 150 µm diameter glass beads (Sigma). The mixture was vigorously shaken three times at max speed (6.5 m/s) for 30 s in a Fastprep homogenizer (Thermo Scientific) to disrupt bacteria. RNA was extracted with chloroform-isoamyl alcohol (24:1) and centrifuged at 16,000 x *g*. The aqueous layer was removed, and total RNA was then precipitated at 4°C overnight with isopropanol and centrifuged for 10 min. The RNA pellet was washed with 75% ethanol, centrifuged, and air-dried. Following suspension in DEPC water, RNA purification was performed using an RNeasy Kit (Qiagen) and TURBO DNase (Ambion) to remove genomic DNA. The lack of amplification confirmed the removal of DNA after 35 cycles employing RNA as template and controls with no RNA template and no reverse transcriptase. The concentration and purity of RNA were assessed in the NanoDrop equipment (ND-1000; Thermo Scientific), and its integrity was determined in the Agilent 2100 bioanalyzer (Agilent). RNA with absorbance ratios at 260/280 nm and 260/230 nm in the range of 1.9 to 2.2 and an RNA Integrity Number (RIN) above 7.0 were considered to use RNA samples for RT-qPCR experiments. cDNA synthesis reaction was realized with 1.0 µg of RNA using the RevertAid First Strand Synthesis kit (Thermo Scientific).

### Quantitative PCR

The LightCycler 480 instrument (Roche) was employed to perform quantitative PCR. Quantification was conducted with specific primers (**Table 1**), which were previously designed with the Primer3plus tool (Untergasser et al., 2007), and by using the SYBR Green I Master mix (Roche). Absolute gene expression was determined by interpolating Ct values to standard curves for each pair of primers, which were obtained using 10-fold dilutions of known copies of *Mtb* H37Rv genomic DNA. Fold change in gene expression was calculated by dividing absolute expression values by the expression of the condition used as a control. 16S rRNA (*rrs*) was used as a reference gene for normalization of qPCR, which was previously tested to be optimal for this purpose by obtaining a standard curve according to 10-fold dilutions of *Mtb* H37Rv genomic DNA (10^3^, 10^4^, 10^5^, 10^6^ and 10^7^ copies). Ct values were interpolated to the standard curve to obtain absolute quantification in copies/µg RNA. The 16S rRNA (*rrs*) gene expression remained unaffected in all conditions tested (**Supplementary Figure S1**).

**Table 1:** Primers for qPCR amplification.

Gene	Sequence (5’ to 3’)	Amplicon size (bp)
***rrs* (16S rRNA)**	F: CTCACCCGTTCGCCACTCG R: CACTGGTGCCTCCCGTAGG	110
**Rv3654c (*tadF*)**	F: TGATCTGGCTTCGTTAGCCG R: GTCCACCACCCTGCACTG	121
**Rv3655c (*tadE*)**	F: AGGTGCGCTGTATCGACG R: ACAGTGGCGACCACAAACTC	148
**Rv3656c(*flp*)**	F: TGATCACCATGTTTCGTGTACT R: GCGGACACAATGGAATCCCC	157
**Rv3657c (*tadC*)**	F: CACGATGCGCAGACCGAT R: ACACACAGAAACGCCGGTAA	200
**Rv3658c (*tadB*)**	F: AGCTGGTGGTGGGTGAA R: CACGCATCAAACTGGCTAT	252
**Rv3659c (*tadA*)**	F: TGGGTGGACGGTCAACT R: GCACACGATCCGCTCAT	300
**Rv3660c (*tadZ*)**	F: CTGGGCGGCTGCCATAA R: CACCGCCGACCAATTCA	328

### Synthesis of Flp-derived peptide and antibody production

A predicted immunogenic region of Flp was chosen from the protein sequence in the publicly available *Mtb* H37Rv Tuberculist genome database (Cole et al., 1998). The amino acid sequence TGDSIVSALNR contained between residues 48-58 of the Flp pilin (Rv3656c) was synthesized at Zymed laboratories. The peptide sequence was blasted against bacterial and eukaryotic protein sequences (NCBI) and found no hits, except for type IV pilins, as predicted. The peptide was conjugated to a suitable carrier to raise rabbit anti-Flp-peptide antibodies using a proprietary PolyQuik accelerated immunization regimen and TiterMax™ (CytRx) adjuvant (Zymed Laboratories). Rabbit pre-immune and post-immune sera were tested using ELISA to measure peptide-specific IgG titers.

### Interaction of *Mtb* H37Rv with U-937 macrophages

U-937 human monocytes (ATCC CRL-1593.2) were differentiated into macrophages with PMA as described above, and cell monolayers were infected with the mycobacteria and incubated at 37°C under an atmosphere of 5% CO_2_ for 2 h. The monolayers were then washed with 1 mL of HBSS, fixed with 2% formaldehyde in PBS, and permeabilized with 0.1% Triton X-100 in PBS for 20 min before the immunofluorescence reaction described below (Knutton et al., 1989).

### Adherence of *Mtb* H37Rv to A549 cell monolayers

A549 cell monolayers were infected with the mycobacteria and incubated at 37°C under an atmosphere of 5% CO_2_ for 1, 4, and 6 h. The monolayers were washed with 1 mL of HBSS, fixed with 2% formaldehyde in PBS for 20 min, and immune-stained with anti-Flp peptide antibody or the pre-immune serum used as a negative control.

### Immunodetection of Flp

Immunofluorescence experiments were done as previously described (Saldaña et al., 2009; Soria-Bustos et al., 2021). The eukaryotic cells were incubated for 1 h with anti-Flp-derived peptide serum diluted 1:1,000 in PBS with 10% of horse serum (Gibco). After incubation, the samples were rewashed and incubated for 1 h with goat anti-rabbit IgG Alexa-fluor 488 conjugate (Molecular Probes) diluted 1:3,000 in 10% horse serum. Propidium iodide (Molecular Probes) was added to stain the eukaryotic nucleus and bacterial DNA. The samples were washed to remove residual staining, mounted on an anti-fade mounting solution (ThermoFisher) on a glass slide, and viewed under UV light with a Nikon TE 2000S fluorescence microscope.

### *In vitro* peptide polymerization

The synthetic Flp-peptide was dissolved in dimethylsulfoxide (DMSO, Sigma) by adding 1 mg of the dried peptide to 100 μL 1% DMSO (Sigma) and diluted to 1 mL in the following sterile filtered aqueous solutions: 10 mM L-Histidine (Sigma), pH 4.5; dH_2_O, pH 6.5; PBS, pH 7.4; 10 mM Tris-HCl (Sigma), pH 8.5; and 150 mM ethanolamine (Sigma), pH 9.5. The peptide solutions were incubated at 37°C and analyzed after 1, 2, 4, 6, and 18 h post-incubation by negative staining and transmission electron microscopy (TEM). After 18 h, the peptide solutions were suspended by vortexing and applied onto a membrane with a 35,000 molecular weight cut-off (YM-35, Amicon) and centrifuged at 5,000 x *g* for 30 min at room temperature. The retentate was recovered with 1 mL sterile PBS and analyzed by immuno-TEM as described below. One-tenth of the retentate was subjected to protein sequencing and MS/MS QTOF at the University of Arizona Proteomics Core facility. Over 60% of the fiber solution contained the amino acid sequence TGDSIVSALN while approximately 30% of the material analyzed represented the amino acid sequence TGDSIVSALNR.

### Transmission electron microscopy

For TEM, 10 μL of the Flp-derived peptide (1 mg/mL) maintained at 4°C were negatively stained on 300-mesh copper grids with 1% phosphotungstic acid (pH 7.4) and analyzed using a Philips CM12 electron microscope operating at 80 kV at the University of Arizona Electron Microscopy Core Facility. For immunogold labeling experiments, the peptide preparation was incubated with 10 μL of anti-Flp peptide serum (diluted 1:10) followed by goat anti-rabbit IgG 10-nm gold conjugate (1:10) (BBL) as previously described (Saldaña et al., 2009). The specimens were negatively stained and viewed by TEM as described above.

### Statistical analysis

For statistical analysis, One-way ANOVA followed by Tukey´s comparison test was performed using GraphPad Prism 9.0 (GraphPad Software Inc., San Diego, CA, USA). Data represent the mean ± standard deviation (SD), and values of *p*<0.05 were considered statistically significant.

## Results

### Identification of type IV pili genes in *M. tuberculosis*

**Figure 1:**
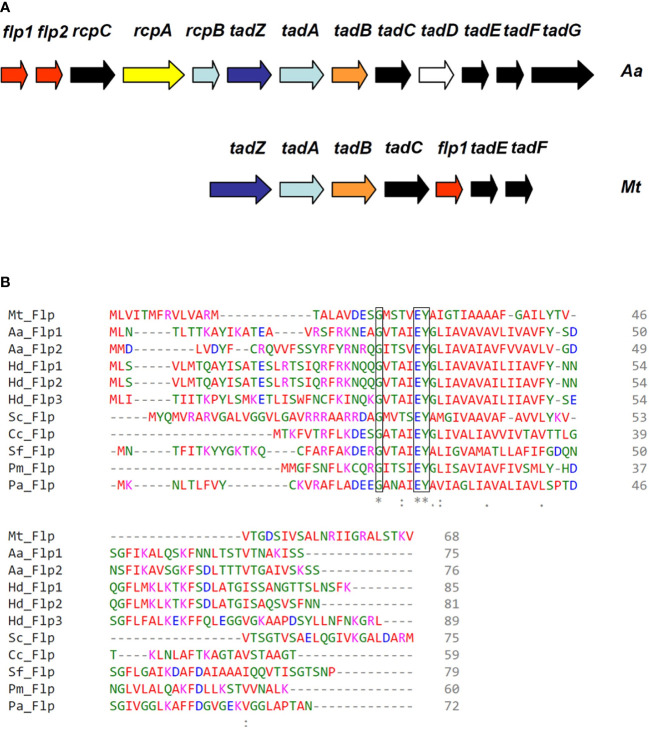
Identification of type IV pili genes of *Mtb*. **(A)** Correlation of *tad*/*flp* gene cluster between *Actinobacillus actinomycetemcomitans* and *Mtb*. Illustrative of the tight adherence (*tad*) locus in *A actinomycetemcomitans Aa* and *Mtb* (Mt). Pilin encoding genes are indicated in red. **(B)** Multiple sequence alignment of Flp type IVB prepilins. *Mtb* H37Rv ORF Rv3656c encodes for a type IVB prepilin. The invariably conserved glycine just preceding the putative cleavage site of the leader peptide, and the glutamate-tyrosine pair at position five of the mature pilin are boxed. The asterisk (*), colon (:), and dot (.) indicate identical, conserved, and semi-conserved amino acids among all aligned sequences. All predicted Flp pilins have an invariable glutamate-tyrosine pair of amino acids. *Haemophilus ducreyi* (Hd), *Streptomyces coelicolor* (Sc), *Caulobacter crescentens* (Cc), *Shigella flexneri* (Sf), *Pasteurella multocida* (Pm), *P. aeruginosa* (Pa). Bacteria containing multiple Flp prepilins are indicated with numeric designations.

The *tad*/*flp* locus, which codes for the assembly machinery of the type IV pili class B in the Gram-negative organism *Actinobacillus actinomycetemcomitans*, is present in the genomes of a broad range of bacterial pathogens, including *Mtb* H37Rv (**Figure 1A**) (Tomich et al., 2007). The genomic organization of the *tad/flp* locus in *Mtb* appears conserved across several high GC actinobacteria, including *Corynebacterium* and *Micrococcus* sp. (Angelov et al., 2015). A conserved Flp pilin amino acid sequence was found in *Mtb* H37Rv, which consists of a specific glycine residue that precedes the putative leader peptide cleavage site and a hydrophobic region containing a glutamate-tyrosine pair of amino acids (**Figure 1B**). The alignment of multiple known and predicted Flp prepilin sequences shows highly conserved features of Flp (**Figure 1B**). Of note, all but one Flp prepilin polypeptide sequence in the multiple sequence alignment is derived from Gram-negative bacteria that are phylogenetically very distant from *Mtb* (**Figure 1B**). In *Mtb* H37Rv the Rv3656c gene is the *flp-*homolog predicted ORF that encodes a polypeptide of 68 amino acids and 6 kDa of molecular mass (Cole et al., 1998). Unlike other Flp pilins, the N-terminal of the *Mtb* Flp protein contains a methionine residue, similar to the Class B type IV pilin TcpA of *Vibrio cholerae* (Taylor et al., 1987).

### The *Mtb tad/flp* locus is located on a genomic island

**Figure 2:**
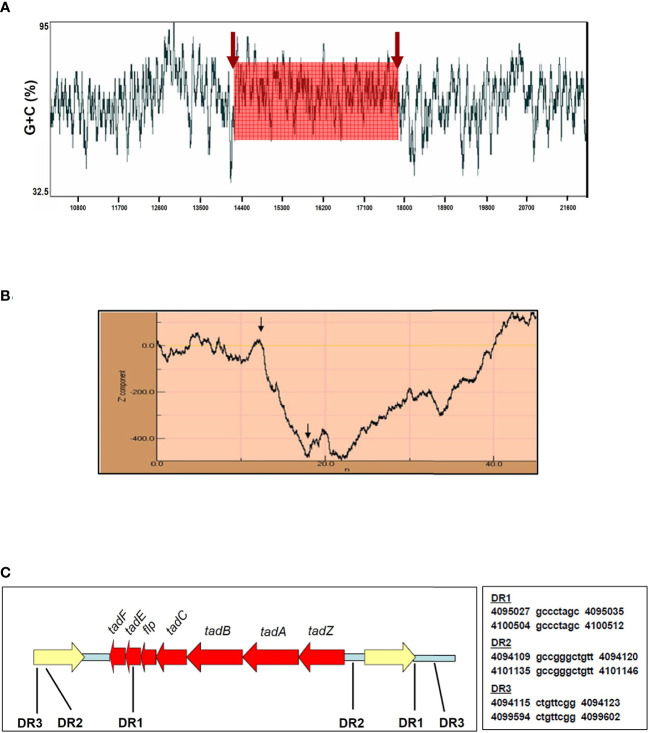
The *flp* locus displays hallmarks of lateral gene transfer. **(A)** The *flp* genes are in a DNA region with a higher G+C content (70%) than the mean G+C content of the *Mtb* H37Rv genome (65%) as determined using a window size of 40 bp. **(B)** Z curve analysis of *Mtb* H37Rv chromosomal section neighboring *flp* genes. Arrows indicate the borders of the *flp* locus in *Mtb* H37Rv. An abrupt decrease in the Z curve shows an increase in G+C content along the DNA sequence, while a sudden increase in the Z curve shows an A+T-rich region. The *tad*/*flp* locus is a chromosomal region, which has a G+C content higher than the neighboring DNA regions. The numbers along the x-axis refer to the input DNA sequence in Kbp. **(C)** Illustrative of the 8,000 bp sequence containing the *tad*/*flp* locus (red) and neighboring genes (yellow) in *Mtb* H37Rv. Direct repeats (DR) in relation to *tad*/*flp* related genes are indicated. The presence of multiple DR neighboring the *tad*/*flp* genes in *Mtb* implies that the acquisition of type IV pili genes was by a horizontal gene transfer event. Arrows in **(A)** and **(B)** indicate the boundaries of the *flp* locus within the *Mtb* chromosome.

Horizontal gene transfer is the acquisition of foreign DNA that typically enhances the fitness of bacteria allowing them to survive, replicate, and adapt to different niches (Hacker and Kaper, 2000). This foreign DNA often contains a unique G+C signature distinct from the host genome, commonly used as a molecular tag of horizontal transfer. In *A. actinomycetemcomitans*, the production of Flp pili correlates with the existence of a widespread colonization island acquired by horizontal gene transfer (Planet et al., 2003). Using the sliding-window G+C content measurement it was determined that *Mtb* H37Rv contains a DNA region with a content of 70% G+C corresponding to the *tad*/*flp* locus location (**Figure 2A**). This finding could be significant due to that the genome of *Mtb* has a G+C content of 65% and it is known that distributive conjugal transfer of genomic DNA occurs in *Mycobacterium* (Gray and Derbyshire, 2018; Clark et al., 2022).

The Z curve method is employed to determine G+C content by performing a windowless procedure with a resolution to a single base, where an A+T rich region and a G+C rich region in the DNA sequence are indicated by a rise and by a drop in the curve, respectively (Zhang et al., 2001). Hence, a significant difference in the curve might suggest the foreign DNA sequence’s horizontal transfer from other bacteria. The resulting Z curve for a 45-kb region of *Mtb* H37Rv genomic DNA surrounding the *tad*/*flp* locus displays an abrupt drop in the curve (**Figure 2B**). This change represents a sudden increment in the G+C content along the DNA sequence. The border of the noticed downward spike coincides accurately with the position of the *tad/flp* locus in the *Mtb* H37Rv chromosome (**Figure 2B**). These results provide compelling evidence that *Mtb* acquired type IV pili genes horizontally from another microorganism.

Direct repeats (DR) in the sequence flanking the acquired DNA or genomic island is another hallmark of horizontal gene transfer (Hacker and Kaper, 2000). At least three DR ranging from 8–11 bp in length in the immediate vicinity of the *tad/flp* genes were found by analyzing 3 kb of upstream and downstream flanking sequences (**Figure 2C**). All three DR were located within 1 kb of the *tad*/*flp* locus. Taken together, the divergent G+C content of the *tad/flp* locus and the presence of multiple direct repeats strongly suggest the occurrence of the insertion of horizontal acquired DNA into the *Mtb* chromosome.

### Growth of *Mtb* at stationary phase induces elevated levels of expression of Flp pili genes

**Figure 3:**
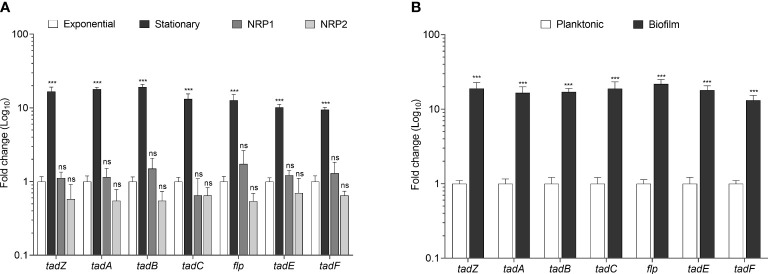
Transcriptional expression of *tad/flp* genes in *Mtb* growing at different growth phases. Fold change expression determined in *Mtb* growing at exponential, stationary, NRP1 and NRP2 phases **(A)** and during planktonic culture and biofilm formation **(B)**. Statistically significant relative to exponential phase **(A)** to planktonic culture **(B)**: ***p<0.001, ns: not significant.

Next, we sought to investigate if the Flp pili genes present in *Mtb* are transcriptionally activated. Thus, we quantitated the expression of the *tad/flp* genes in *Mtb* H37Rv grown in stationary, NRP1, and NRP2 phases in comparison to growth in the exponential phase using RT-qPCR with specific primers (**Table 1**). The expression of all the seven *tad/flp* genes during growth at the stationary phase was more than 10-fold higher than in the exponential phase. The expression levels during early hypoxia or microaerophilic condition (NRP1) were remarkably similar with respect to the exponential phase, while during late hypoxia or anaerobic condition (NRP2) slightly decreased 1.66-fold; however, in both hypoxia conditions, the expression of the seven genes was not statistically significant with respect to the exponential phase. (**Figure 3A**).

### Relative gene expression during biofilm formation

Growing evidence strongly indicates that *Mtb* produces robust biofilms (Ojha et al., 2008; Ramsugit et al., 2013; Esteban and García-Coca, 2017; Richards et al., 2019). We sought to determine the expression of *tad/flp* pili genes during the formation of biofilms in *Mtb* H37Rv. The expression of *tad*/*flp* genes was 17.8-fold higher during biofilm formation than in planktonic culture (**Figure 3B**).

### Expression of *tad/flp* pili genes during interaction of *Mtb* with U-937 macrophages and A549 pneumocytes

**Figure 4:**
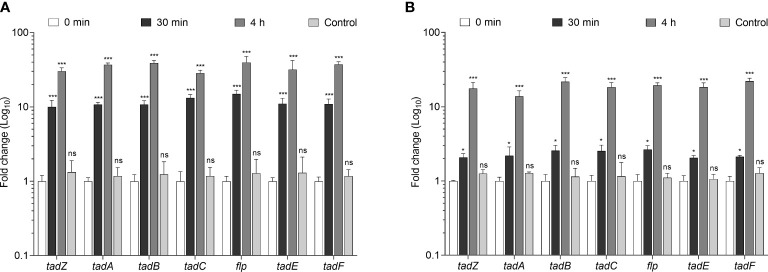
Transcriptional expression of *Mtb tad/flp* genes upon interaction with macrophages and human alveolar epithelial cells. **(A)** The human macrophage cell line U-937 and **(B)** human alveolar epithelial basal cell line A549 were infected with *Mtb* H37Rv for 0 min, 30 min, and 4h. As control, RPMI-1640 medium alone was used for incubation of *Mtb* at 37°C for 4h. Data are the mean of three different assays accomplished in triplicate with SD values. Normalization of expression was conducted by using 16S rRNA gene (*rrs*). Statistically significant relative to time zero: ***p<0.001, *p<0.05, ns, not significant.

During pathogenesis, type IV pili of many bacteria are involved in attachment to eukaryotic host cells (Craig et al., 2004). We were then interested in determining whether *Mtb* would express *tad/flp* genes during infection of host cells. To this aim, we infected U-397 macrophages and A549 pneumocytes with *Mtb* H37Rv for 30 min and 4 h. In infected U-937 macrophages, an average 11.7- and 34.8-fold increase in gene expression was observed for all the *tad/flp* genes at 30 min and 4 h post-infection, respectively, with respect to time zero. As a control, *Mtb* was cultured for 4 h in RPMI medium alone. The expression levels were remarkably similar to the time zero (**Figure 4A**). Expression of all the seven *tad/flp* genes was 2.3- and 18.8-fold increased during interaction of *Mtb* with A549 pneumocytes at 30 min and 4 h post-infection, respectively (**Figure 4B**). The expression data for the control (RNA obtained from *Mtb* grown in RPMI medium alone and incubated at 37°C for 4 h) were remarkably similar to the time zero.

**Figure 5:**
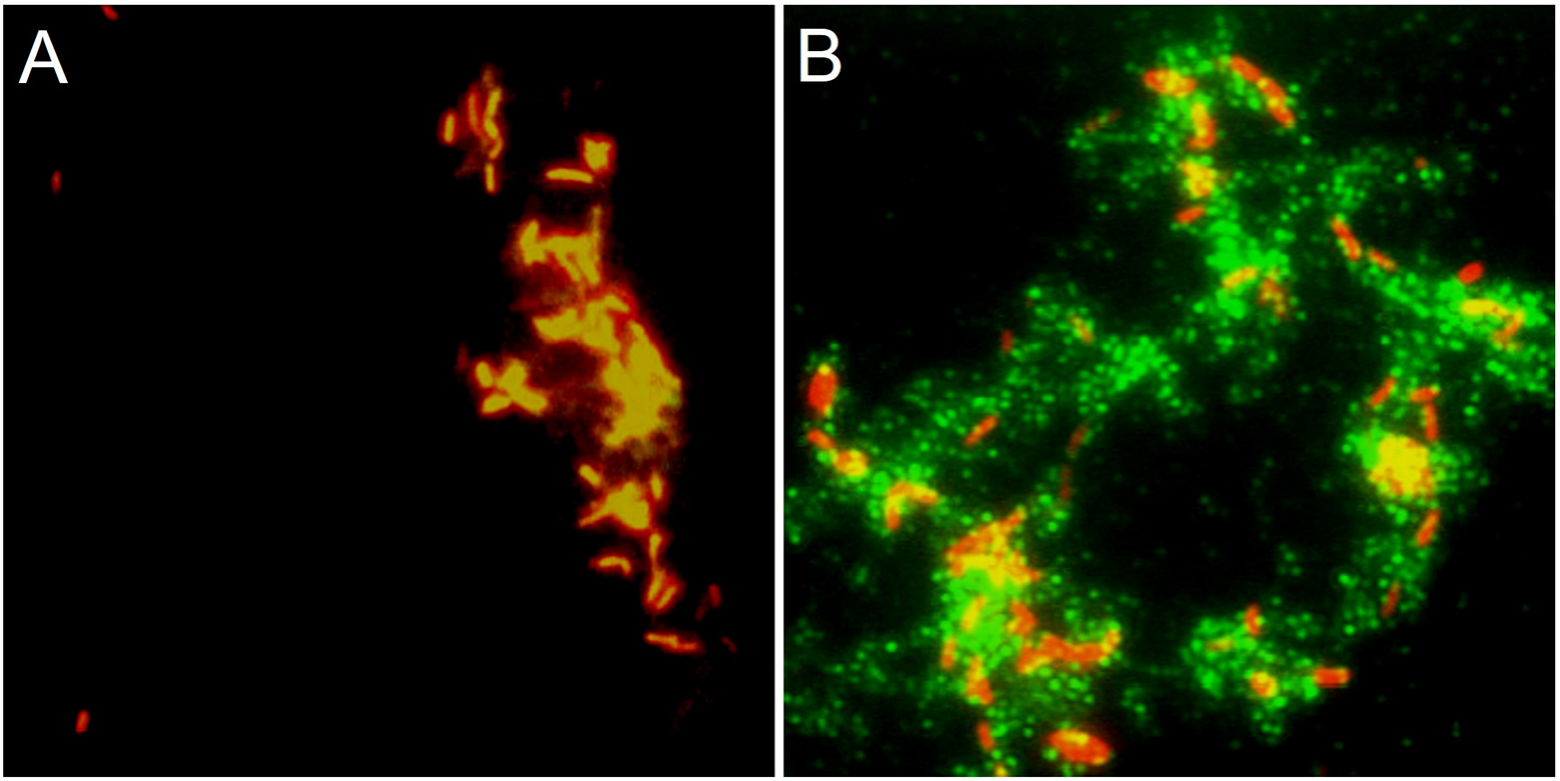
*Mtb* produces Flp. Immunofluorescence microscopy of *Mtb* H37Rv with anti-Flp peptide antibodies. Aliquots of stationary phase *Mtb* grown in standard mycobacteriological media were fixed with formalin onto glass coverslips before being incubated with **(A)** pre-immune or **(B)** anti-Flp peptide sera. Alexa Fluor 488 anti-rabbit IgG conjugate (green) was employed to detect the primary antibody. Propidium iodide was used to label the bacterial cells (red). Samples were incubated with anti-Flp peptide antisera **(B)** and not with pre-immune sera **(A)** from the same rabbit to observe the Flp-specific reaction. Magnifications are 1,000X.

The immunofluorescence microscopy experiments provide additional evidence for producing the Flp antigen on the surface of the mycobacteria. We found that the antibodies raised against a 12-mer peptide derived from the Flp pilin (anti-Flp peptide antibodies) specifically detected Flp associated with the mycobacteria (**Figure 5B**), while the pre-immune serum from the same rabbit did not generate any detectable immunofluorescence reaction (**Figure 5A**).

In all, these results are compelling evidence that *Mtb* Flp is expressed in different conditions of growth and during interaction with eukaryotic cells, as is seen with other bacterial pathogens that manifest this family of organelles (Girón et al., 1991; Nagai et al., 1991; Sonnenberg and Belisle, 1997; Kachlany et al., 2001a). This is the first report showing that *tad/flp* genes are expressed in mycobacteria.

### Flp pili are detectable during the interaction of *Mtb* with eukaryotic cells

**Figure 6:**
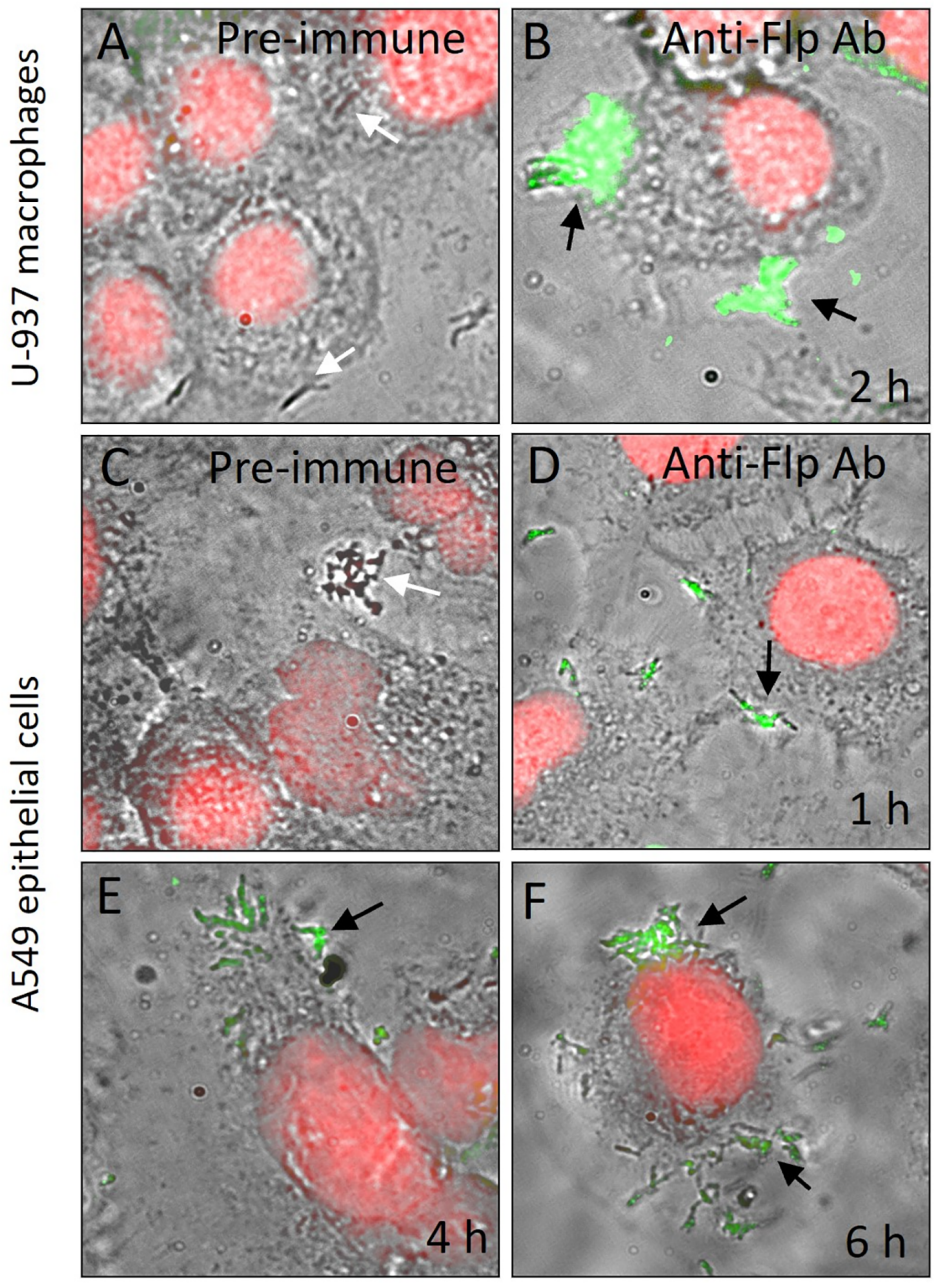
Flp is produced during *Mycobacterium*-host cell interaction. U-937 macrophages **(A, B)** and A549 human epithelial cells **(D–F)** were infected with *Mtb* H37Rv **(A)** for the indicated time periods and then stained with pre-immune serum **(A, C)** or anti-Flp peptide antibodies **(B, D–F)**. Propidium iodide was used to stain the nuclei (red) and anti-rabbit IgG Alexa fluor 488 conjugate (green) was used to detect anti-Flp peptide antibodies. To show bacterial and eukaryotic cells as well as Flp production, phase contrast light microscopy and fluorescence images were merged. Black arrows indicate bacteria showing associated fluorescence and white arrows in panels **(A, C)** indicate bacteria with no fluorescence. At 2 h post-infection of U-937 cells, Flp (black arrows) is detectable on the cell-associated mycobacteria **(B)**. The kinetics of infection of A549 cells followed by immunofluorescence show increasing number of mycobacteria from 1 h to 6 h of infection, which correlates with the level of fluorescence associated with the mycobacteria. The presence of Flp appeared to be concentrated at 6 h post-infection in areas where numerous bacilli are adhering to the epithelial cells **(F)**. Magnifications are 1,000X.

For many bacterial pathogens, the expression of virulence factors is triggered by the attachment to host cells or environmental cues (Esposito et al., 2012; Govender et al., 2014; Mann et al., 2016). We sought to investigate whether *Mtb* H37Rv would produce Flp during adherence to A549 epithelial cells and invasion of U-937 macrophages. To this aim, we infected U-937 macrophages for 2 h and A549 pneumocytes with *Mtb* H37Rv for 1-6 h and monitored the production of Flp probing with anti-Flp peptide antibodies by immunofluorescence microscopy experiments. We found that at 2 h post-infection of U-937 cells, nearly all the macrophage-associated *Mtb* H37Rv bacteria displayed on their surface, specific immunostaining representing the presence of the Flp pilin (**Figure 6B**). The fluorescence visualized was tightly wrapped around the bacteria and appeared on bacteria directly in contact with the eukaryotic cells. The specificity of the immunofluorescence reaction was demonstrated in the negative control consisting of the staining of infected U-937 cells with the pre-immune serum from the same rabbit (**Figure 6A**). The kinetics of infection of A549 cells followed by immunofluorescence show an increasing number of mycobacteria from 1 to 6 h of infection, which correlates with the level of specific fluorescence associated with the mycobacteria (**Figures 6D–F**). Flp appeared to be concentrated at 6 h post-infection in areas where numerous bacilli adhere to the epithelial cells (**Figure 6F**). The pre-immune serum used as a negative control did not produce any detectable reaction (**Figure 6C**), which supports the specificity of the anti-Flp peptide antibody.

### *In vitro* polymerization of the Flp-derived peptide

**Figure 7:**
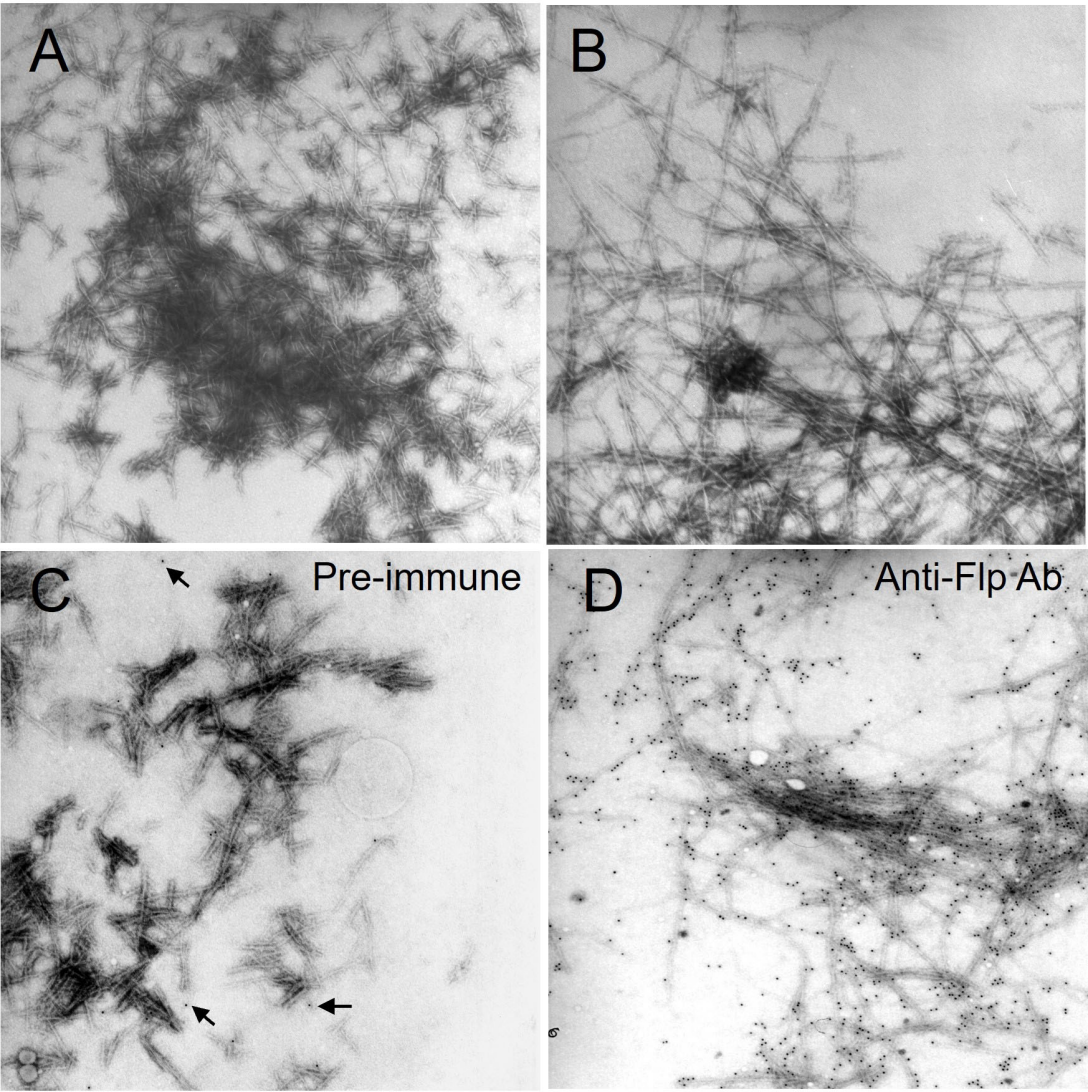
Flp peptide polymerizes *in vitro*. **(A)** At 2h post-incubation of purified Flp-peptide, densely staining aggregates showing short fibers are detectable. **(B)** The fibers appear longer and are more loosely associated after 18h incubation. Afterwards, the samples were recouped and passed through a cut-off filter. The substance retained by the filter was resuspended in PBS before immuno-electron microscopy experiments with either pre-immune **(C)** or anti-Flp peptide sera **(D)**. The localization of the 10 nm colloidal gold particles with the fibers occurs only in the presence of anti-Flp peptide antisera. Original magnifications are 25,000X.

It has been demonstrated that whole or fractions of pilin polypeptides can self-assemble *in vitro* (Guterman et al., 2016). The carboxyl terminus of *A. actinomycetemcomitans* Flp pili is critical for the assembly of full-length Flp fibers (Kachlany et al., 2001b). Thus, experiments were done to determine if an *Mtb* Flp pilin carboxyl terminus peptide could polymerize into pili-like fibers *in vitro*. Interestingly, by negative staining and TEM, we observed that the Flp pilin-derived peptide could assemble into a filamentous structure (**Figure 7**). Aggregates of fibers with an average length of 5 µm and an approximate diameter of 7 nm were observed as early as 2 h post-incubation at pH 4.5 (**Figure 7A**). Like pili produced by bacteria *in vivo*, the aggregates decreased in a time-dependent pattern concomitant with the presence of longer 7 nm diameter fibers (**Figure 7B**). At physiological pH 6.5 and pH 7.4, the same time-dependent pattern of Flp pilin-peptide polymerization was observed. Interestingly, at pH 8.5 and pH 9.5, no fiber formation was observed, indicating that acidic to neutral pH is influential for promoting inter-peptide polymerization or that elevated pH is inhibitory for the observed phenomenon. Further, the fibers were immuno-stained with anti-Flp-peptide antibodies in gold-labeling experiments, as observed by the co-localization of the 10 nm colloidal gold particle conjugate (**Figure 7D**). Pre-immune serum from the same rabbit failed to produce significant gold co-localization with the fibers (**Figure 7C**). To support the notion that the Flp pilin-peptide was auto-assembling into pili-like structures, we pre-incubated Flp monomeric peptide with anti-Flp peptide. The rationale for this was that the presence of anti-Flp pilin antibodies would inhibit the polymerization of the Flp pilin peptide. In support of this, when the Flp-peptide was pre-incubated with the cognate antiserum, the observed fiber formation did not occur (data not shown). The polymerization experiments provide indirect evidence that the *Mtb* Flp type IV pilin homolog is capable of being assembled into a fibrous organelle.

## Discussion

Attachment and invasion of epithelial cells and intracellular living within alveolar macrophages are considered pathogenic traits for *Mtb* (Cianciotto, 2005; Kinhikar et al., 2006; Hickey et al., 2010; Esposito et al., 2012; Lebrun et al., 2012; Nandakumar et al., 2013). Several extracellular molecules of *Mtb* such as the HBHA protein, glycoprotein Apa, enzyme malate synthase, 19-kDa antigen, chaperones DnaK and CPN60.2, and MTP are reportedly involved in the adhesion of the bacteria to lung epithelial cells and tissues, and several cell lines (Diaz-Silvestre et al., 2005; Kinhikar et al., 2006; Alteri et al., 2007; Hickey et al., 2010; Esposito et al., 2012; Lebrun et al., 2012; Kumar et al., 2013; Nandakumar et al., 2013; Govender et al., 2014). The Flp pili are a class of type IV pili which are involved in several virulence-associated traits in many disease-causing bacteria (Planet et al., 2003; Schreiner et al., 2003; Craig et al., 2004). The Flp pili were first described in the periodontal pathogen *A. actinomycetemcomitans*. They were shown to be required for adherence of the bacteria to oral cavity surfaces and subsequent pathogenesis of the periodontal disease (Kachlany et al., 2001b). Flp pili is also required for adherence to human fibroblasts and virulence in humans by *Haemophilus ducreyi*, the causative agent of human chancroid disease (Spinola et al., 2003). The presence in the *Mtb* genome of Flp type IV pili gene homologs (*tadZ, tadA, tadB, tadC, flp, tadE*, and *tadF*) has been reported (Tomich et al., 2007; Imam et al., 2011; Angelov et al., 2015; Ramsugit and Pillay, 2015). In this study, we report the outcome of our investigation on the Flp pili of *Mtb* H37RV directed towards understanding the expression of the pili at different growth phases, during biofilm formation, and during interaction with phagocytic and host epithelial cells. Analysis of the predicted Flp prepilin amino acid sequence reveals characteristics of type IV class B prepilins, which include the invariable glycine preceding the site of signal peptide cleavage and the glutamate-tyrosine pair conserved located in the fifth position of the sequence (**Figure 1B**). To learn about the origin of the Flp locus, we determined the G+C content of this DNA region. The *Mtb* chromosome has a mean range of 65% G+C, in contrast to the *Mtb tad/flip* locus that was found to contain a mean of 70% G+C. This differing G+C content coincided with the boundary of the type IV pili genes. The regions flanking the Flp pili genes were found to contain multiple DR, which are genetic signatures indicative of foreign DNA insertion. *Caulobacter cresentens* and *Pseudomonas aeruginosa* are Gram-negative microorganisms with mean G+C contents of 70% and 68%, respectively, which is a G+C content higher than *Mtb* and also possess Flp pili genes (Planet et al., 2003). BLAST analyzes indicate that this Flp pilus is distantly related to other bacteria, suggesting that the events leading to the horizontal transfer of these genes to *Mtb* occurred some time ago, and the synteny with *tad/flp* genes of Gram-negative bacteria was lost as the mycobacteria evolved. Furthermore, the finding of various DR flanking the *tad/flp* genes in *Mtb* clearly shows that these genes were incorporated into its genome by horizontal gene transfer. These are compelling data that indicate that the Flp pili genes are contained in a genomic island and highlight the possibility that *Mtb* acquired these type IV pili genes by horizontal gene transfer. This is reasonable as it has been shown that horizontal transfer of genomic DNA occurs in *Mycobacterium* (Gray et al., 2013), which can involve ESX secretion systems (Gray et al., 2016) or even the *tad/flp* locus itself, as seen in the high G+C actinobacteria *Micrococcus luteus* (Angelov et al., 2015). Please change this sentence to: The origin of the Flp-containing genomic island in *Mtb* remains to be determined. Horizontal transfer of DNA encoding toxins, antimicrobial resistance genes, adhesins, iron uptake systems, pili, and various secretion systems represents a major evolutionary force and advantage for microbial pathogens to adapt to different hosts and overcome the immune system (Hacker and Kaper, 2000). The presence of an Flp-containing genomic island in *Mtb* would presumably increase this bacterium’s fitness and ability to colonize host tissues.

An important question to address next was if the Flp pili genes were expressed. The *tad/flp* genes of *Mtb* were over-expressed in the stationary phase, which agrees with studies reported in other bacteria such as *P. aeruginosa*, *Thermus thermophilus*, and *Proteus mirabilis*, where also it was observed a significant over-expression of type IV pili in the stationary phase (Bernard et al., 2009; Kuan et al., 2014; Salzer et al., 2014). It is known that most fimbrial genes increase their expression in the stationary phase. Such an increase has been attributed to the fact that most bacteria respond to environmental stress signals to survive. During the stationary phase, there is a lack of nutrients and accumulation of toxic substances that generate mechanisms of persistence and protection in bacteria, mechanisms in which the pili have been shown to participate (Gefen et al., 2014). One mechanism for bacterial persistence is mediated by filamentous structures such as type IV pili causing the formation of bacterial microcolonies and biofilm (Craig and Li, 2008). Furthermore, during the stationary phase, the number of bacteria increases therefore the bacteria-bacteria contact might stimulate the over-expression of the *tad/flp* genes in *Mtb*, such as was reported in other bacteria, where it was shown that during the stationary phase the type IV pili were overexpressed due to bacterial auto-aggregation (Ramer et al., 2002; Nwoko and Okeke, 2021). The expression of the *Mtb tad/flp* genes was also analyzed using the *in vitro* model of latency induced by hypoxia (Wayne and Hayes, 1996). During the NRP1 and NRP2 phases, the expression of *tad/flp* genes was remarkably similar with respect to the exponential phase. These observations coincided with studies carried out in *V. cholerae* where the type IV pili TCP maintained its expression in anaerobiosis as in aerobiosis (Krishnan et al., 2004). Our results suggest that the Flp pili of *Mtb* may not participate during dormancy.

Previous studies have shown that type IV pili are overexpressed upon contact with macrophages and pneumocytes (Boekema et al., 2004; Chakraborty et al., 2008; Piepenbrink et al., 2016; Okaro et al., 2022). In this study, the *Mtb tad/flp* genes were significantly up-regulated upon contact with U-937 macrophages and A549 pneumocytes, suggesting that contact between bacteria and macrophages could trigger the transcription and subsequent production of Flp pili to facilitate adherence of the bacteria to host cells.

The ability of *Mtb* to survive within macrophages symbolizes the central paradigm of its pathogenesis (Vergne et al., 2005; Danelishvili et al., 2010; Lee et al., 2011; Jamwal et al., 2016; Bade et al., 2021). Despite many years of research, the molecular mechanisms related to adherence and invasion of the macrophage by *Mtb* are not fully elucidated. A key aspect of this research was the finding that the Flp pili are expressed intracellularly when the mycobacteria infect macrophages. To accomplish this, we synthesized a 12-residue peptide derived from the Flp pilin protein, which was used as an antigen to raise antibodies against the Flp. These antibodies were used as probes to detect the presence of Flp pili on the surface of bacteria infecting macrophages at different time points. The fact that Flp pili are present in the intracellular niche of the macrophage suggests that pili promote interbacterial interactions, similar to the contribution of type 1 pili in the formation of intracellular bacterial communities produced by uropathogenic *E. coli* in bladder epithelial cells (Wright et al., 2007). While we did not successfully visualize the Flp pili by TEM, the immunofluorescence experiments performed on infected macrophages strongly support the expression data.

Interestingly, we found that a peptide derived from the Flp pilin polymerizes *in vitro* into fibers resembling pili over a pH range of 4.5 to 7.4. These pili-like fibers were stained with anti-Flp-peptide antibodies. The apparent pH-dependence for the Flp-peptide polymerization could have implications regarding the role of Flp pili for *Mtb* pathogenesis because an important mycobacterial survival signal within the host is the acidic pH of the phagosomal vacuole.

The potential contribution of the *tad/flp* genes to *Mtb* virulence was recently investigated (Mann et al., 2016). *tad* mutants of *Mtb* strain Erdman were not altered during biofilm development with respect to the wild-type strain. Similarly, *tad* mutants were not attenuated for pulmonary and splenic burden in C57Bl/6 mice at various time points post-infection. The authors concluded that the Flp pilin is not required for the ability of *Mtb* to form community associations in culture or to infect a mouse model. Future studies are needed to elucidate the precise role of Flp pili in the pathogenic scheme of *Mtb.*

## Data availability statement

The original contributions presented in the study are included in the article/**Supplementary Material**. Further inquiries can be directed to the corresponding authors.

## Author contributions

CA, RF, JG-M, JT, MC, MA, and JG conceived and designed the study. CA, NR-S, and MA performed the experiments. CA, NR-S, MD, JG-M, JS-B, CM-B, MC, JY-S, YM-L, JT, RF, JG, and MA analyzed the data. CA, NR-S, JG-M, JG, and MA wrote the manuscript. All authors contributed to the article and approved the submitted version.

## Acknowledgments

We thank Lizbel León-Solís and Cecilia Helguera-Repetto for technical assistance. We thank the Vice-Rectoría de Investigación (BUAP) for support. RF was the PhD Thesis advisor of CA.

## Conflict of interest

The authors declare that the research was conducted in the absence of any commercial or financial relationships that could be construed as a potential conflict of interest.

## Publisher’s note

All claims expressed in this article are solely those of the authors and do not necessarily represent those of their affiliated organizations, or those of the publisher, the editors and the reviewers. Any product that may be evaluated in this article, or claim that may be made by its manufacturer, is not guaranteed or endorsed by the publisher.
