# Supplementary figures and images for: The Flp type IV pilus operon of Mycobacterium tuberculosis is expressed upon interaction with macrophages and alveolar epithelial cells
Source: Front Cell Infect Microbiol. 2022 Sep 20;12:916247. doi: 10.3389/fcimb.2022.916247 (PMC9531140; doi:10.3389/fcimb.2022.916247)

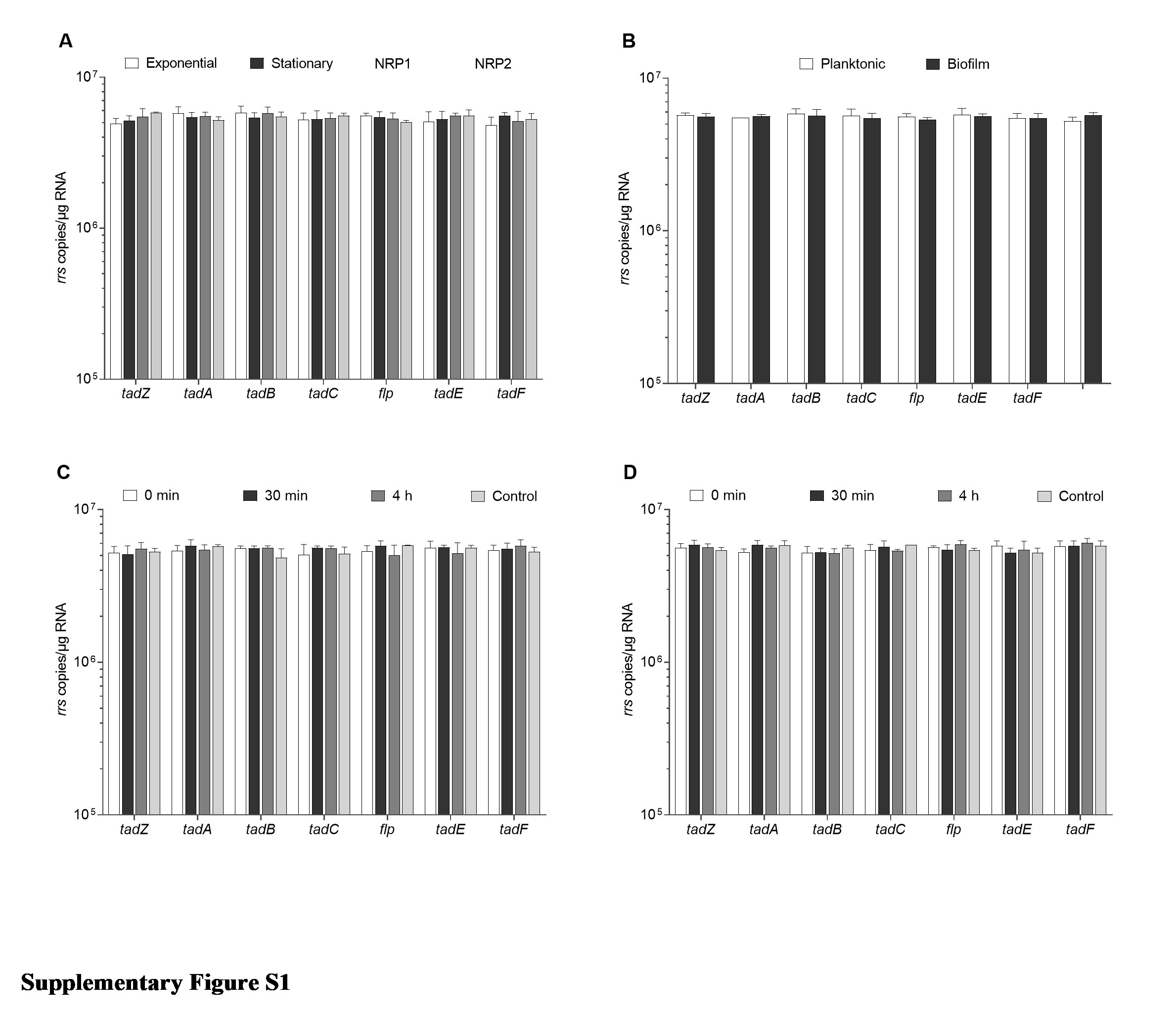

Supplement: Supplementary file 1 [file Image_1.tif]
